# Deep learning based feature-level integration of multi-omics data for breast cancer patients survival analysis

**DOI:** 10.1186/s12911-020-01225-8

**Published:** 2020-09-15

**Authors:** Li Tong, Jonathan Mitchel, Kevin Chatlin, May D. Wang

**Affiliations:** 1 Department of Biomedical Engineering, Georgia Institute of Technology and Emory University, 313 Ferst Dr. NW, Atlanta, 30332 USA; 2 Department of Biomedical Engineering, Georgia Institute of Technology, 313 Ferst Dr. NW, Atlanta, 30332 USA

**Keywords:** Multi-omics integration, Breast Cancer, Survival analysis, Deep learning

## Abstract

**Background:**

Breast cancer is the most prevalent and among the most deadly cancers in females. Patients with breast cancer have highly variable survival lengths, indicating a need to identify prognostic biomarkers for personalized diagnosis and treatment. With the development of new technologies such as next-generation sequencing, multi-omics information are becoming available for a more thorough evaluation of a patient’s condition. In this study, we aim to improve breast cancer overall survival prediction by integrating multi-omics data (e.g., gene expression, DNA methylation, miRNA expression, and copy number variations (CNVs)).

**Methods:**

Motivated by multi-view learning, we propose a novel strategy to integrate multi-omics data for breast cancer survival prediction by applying complementary and consensus principles. The complementary principle assumes each -omics data contains modality-unique information. To preserve such information, we develop a concatenation autoencoder (ConcatAE) that concatenates the hidden features learned from each modality for integration. The consensus principle assumes that the disagreements among modalities upper bound the model errors. To get rid of the noises or discrepancies among modalities, we develop a cross-modality autoencoder (CrossAE) to maximize the agreement among modalities to achieve a modality-invariant representation. We first validate the effectiveness of our proposed models on the MNIST simulated data. We then apply these models to the TCCA breast cancer multi-omics data for overall survival prediction.

**Results:**

For breast cancer overall survival prediction, the integration of DNA methylation and miRNA expression achieves the best overall performance of 0.641 ± 0.031 with ConcatAE, and 0.63 ± 0.081 with CrossAE. Both strategies outperform baseline single-modality models using only DNA methylation (0.583 ± 0.058) or miRNA expression (0.616 ± 0.057).

**Conclusions:**

In conclusion, we achieve improved overall survival prediction performance by utilizing either the complementary or consensus information among multi-omics data. The proposed ConcatAE and CrossAE models can inspire future deep representation-based multi-omics integration techniques. We believe these novel multi-omics integration models can benefit the personalized diagnosis and treatment of breast cancer patients.

## Background

Breast cancer is the most common type of cancer in females worldwide. In 2018, breast cancer constituted over 25% of about 8.5 million new cancer diagnoses in female patients [1]. This prevalence pattern is found in the US as well, where women have over a 12% risk of being diagnosed with breast cancer in their lives, and breast cancer cases are expected to encompass about 30% of new cancer cases [2]. While the principal risk factor for breast cancer is age, it is known that selected gene mutations account for about 10% of all breast cancer cases [3]. Research into prognostic genomic biomarkers beyond mutational status is ongoing and may offer insights into disease mechanisms and new therapies. Breast cancer maintains the second-highest mortality rate for cancers in females at about 13% [2]. Survival rates for breast cancer are typically measured by 5-year post-diagnosis survival. The 5-year survival rate is 90% when all stages are combined [4]. If each cancer stage is considered separately, the 5-year survival rate is 99% for localized breast cancer and drops to 85 and 27% for regionally and distantly spread cancer, respectively.

Public multi-omics datasets such as The Cancer Genome Atlas (TCGA) [5] have greatly accelerated the research for cancer study [6], including accurate cancer grading, staging, and survival prediction [7–9]. The cancer survival analysis can be categorized into binary classification or risk regression. In a binary classification task, the patients are typically split into a short-survival group and a long-survival group based on a predefined threshold (e.g., 5 years). While in risk regression studies, a risk score is calculated for each patient, typically with the Cox proportional hazards model [10] and its extensions.

Various models have been developed for survival prediction in large and heterogeneous cancer datasets. For example, Zhao et al. have tested various classification algorithms to predict 5-year breast cancer survival by integrating gene expression data with clinical and pathological factors [11]. Authors find that various classification methods (e.g., gradient boosting, random forest, artificial neural networks, and support vector machine) have similar accuracy and area under the curve (AUC) of 0.72 and 0.67, respectively. This study demonstrates that classification methods may not matter as much as the quality of the data itself [11]. Goli et al. have developed a breast cancer survival prediction model with clinical and pathological data using support vector regression and find similar positive results [12]. This study has established the use of support vectors as a promising route in survival prediction with an imbalanced dataset. Similarly, Gevaert et al. have integrated microarray gene expression data with clinical data using Bayesian Networks and achieved a maximum AUC of 0.845 [13]. This study shows that incorporating both data modalities improved predictions beyond either clinical or gene expression alone. Sun et al. have created 5-year breast cancer survival prediction models using genomic data (e.g., gene expression, copy number alteration, methylation, and protein expression) coupled with pathological imaging data also from TCGA. The authors utilize multiple kernel learning to enact feature-level integration of all data. Their multi-omics model, excluding imaging data, has an AUC of 0.802 ± 0.032. When incorporating the imaging data, the AUC goes up slightly to 0.828 ± 0.034 [14]. Ma et al. have applied factorization autoencoder to integrate gene expression, miRNA expression, DNA methylation, and protein expression for progression-free interval event prediction and achieve an AUC of 0.74 on bladder cancer and an AUC of 0.825 on brain glioma [15].

Instead of binary classification, the survival risk regression aims to predict the expected duration of time until one or more events happen by modeling the time to event data. The proportional hazards model assumes the covariates are multiplicatively related to the hazard [16]. Assuming the proportional hazards assumption holds, the Cox proportional hazards model can estimate the effect parameters without considering the hazard function [10]. Recently, the Cox proportional hazards model has been extended by deep neural networks. For example, Deep Surv [17] and Cox-Time [18] replace the linear relationship in the Cox proportional hazards model with non-linear neural networks. In addition, *L*_1_ and *L*_2_ regularization terms have been utilized on the network parameters to reduce the over-fitting of the models. The survival risk regression model has also been applied to multi-omics data. For example, Huang et al. have developed a Cox-proportional hazards model based multi-omics neural network for breast cancer survival regression [19].

In our previous study [20], we have built a transnational pipeline for overall survival prediction of breast cancer patients by decision-level integration of multi-omics data (e.g., gene expression, DNA methylation, miRNA expression, and copy number variations (CNVs)). However, many right-censored samples have been discarded to enable binary classification. In this study, we extended the work by replacing the binary survival classification with survival risk regression to make the most of the TCGA dataset. We hypothesize there are both complementary and consensus information in the multi-omics data. To utilize the complementary and consensus information among multi-omics data, we replace the decision-level integration with deep learning-based feature-level integration. The remainder of the paper is structured as follows: in section 2, we first describe the simulated two-view data from the Modified National Institute of Standards and Technology (MNIST) database and multi-omics breast cancer (BRCA) data from the TCGA database (referred as TCGA-BRCA hereafter). We then present the proposed methods for multi-omics data integration by utilizing the complementary information and consensus information among modalities. In section 3, we present the results of the baseline models and proposed models on both MNIST simulated data and TCGA-BRCA multi-omics data. We will discuss the results and conclude the current work in section 4 and section 5, respectively.

## Methods

### Simulated multi-view MNIST dataset

To validate the proposed feature-level integration network, we simulate the multi-modality data from the Modified National Institute of Standards and Technology (MNIST) database. The MNIST database consists of 60,000 training samples and 10,000 testing samples. Each sample in the MNIST database is a 28 × 28 image of a single hand-written digit from 0 to 9. The goal is to train a multi-class classifier to predict the digit from the input image.

**Fig. 1:**
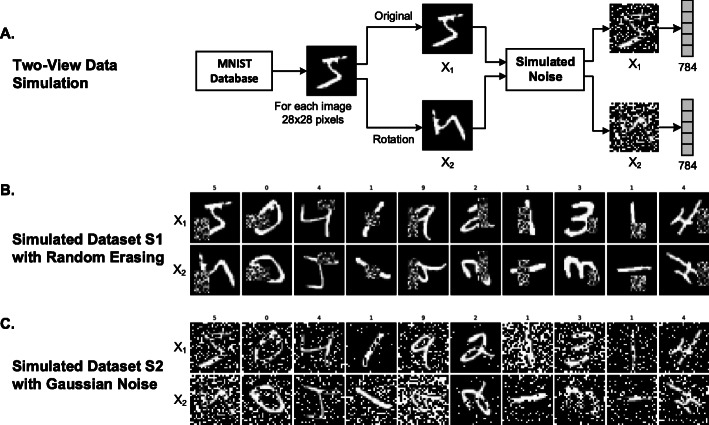
Simulation two-view data from the MNIST database. **a** Pipeline for simulation of two-view data from the MNIST database. **b** Simulated dataset *S*_1_ with random erasing noise. **c** Simulated dataset *S*_2_ with Gaussian noise

We simulate two-views of each hand-written digit image from the MNIST database (Fig. 1a). The first view (*X*_1_) is the original image from the MNIST database, while the second view (*X*_2_) is the corresponding rotated image (90-degree counter-clockwise rotation). We further simulate noises for the data because the task is easy even for single-view data. We have simulated two kinds of noises and apply them to both views of the hand-written digit images: random erasing (Fig. 1b) and pixel-wise Gaussian noise (Fig. 1c). We flatten the image to a vector with a length of 784 as the final input to deep neural networks.

### TCGA-BRCA breast cancer multi-omics dataset

TCGA database [5] is a public database containing genomic data for over 20,000 paired cancer and normal samples from 33 cancer types. In this study, we are using TCGA-BRCA, which has 1060 patients with all four types of -omics data (e.g., gene expression, miRNA expression, DNA methylation, and CNVs) and survival information (see Supplementary Material Section S2). Table 1 contains information about the four omics data types. For gene expression, the number of features includes different isoforms for each gene and some non-coding RNA transcripts. The DNA methylation beta value ranges from 0 to 1, where a beta value of 0 means that no methylation is detected for that probe, while a 1 means that the CpG was always methylated. For CNV features, “Gain” means more copies of a gene than normal, while “Loss” means fewer copies of a gene than normal. More details for the TCGA multi-omics data can be found in Supplementary Material Section S1.

**Table 1:** Overview of four omics data modalities

**Data Modality**	**Gene Expression**	**DNA Methylation**	**miRNA Expression**	**Copy Number Variation**
**Measures**	Fragments per kilobase of transcript per million mapped reads (FPKM)	Beta Value	Reads per million mapped reads (RPM)	Gain/Loss/Neutral
**Dynamic** **Range**	Continuous [0, 3,823,803,664.0]	Continuous [0, 1]	Continuous [0, 679,286.5]	Discrete {“Loss”: − 1,” Neutral”: 0,” Gain”:1}
**Feature** **Name**	Ensembl Gene ID	cg probe identifier	miRNA identifier	Ensembl Gene ID
**# of Features**	60,483	25,978	1881	19,729

**Fig. 2:**
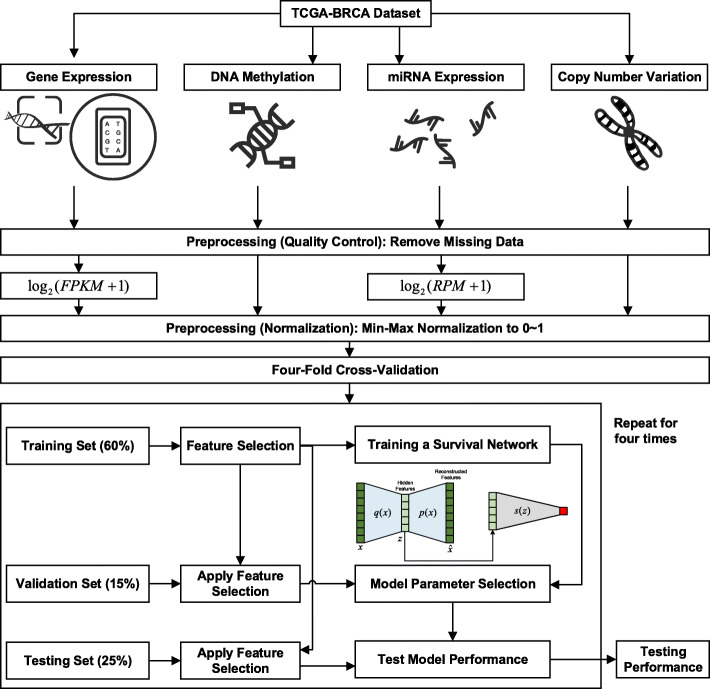
Overall pipeline for survival analysis. We obtain multi-omics data (i.e., gene expression, DNA methylation, miRNA expression, and copy number variation) for breast cancer patients from the TCGA-BRCA database. The multi-omics data are preprocessed and normalized to a range of 0 to 1. We then apply four-fold cross-validation and split the data into a training set (60%), validation set (15%), and testing set (25%) in each fold. We train the feature selection or dimension reduction step and the survival networks using the training set and apply them to the validation set for parameter selection and the testing set for performance reporting

The overall pipeline for multi-omics survival analysis is presented in Fig. 2. Quality control and preprocessing are essential for making sense of multi-omics data. To get rid of the low-quality features, we remove features with missing data. For the gene expression and miRNA expression data, we also apply a log transform log_2_(*X* + 1) to the features, where *X* is the FPKM for gene expression and RPM for miRNA expression. We then apply min-max normalization to scale all four data modalities to a range of 0 to 1. After the quality control and normalization, we apply a stratified four-fold split of the data into a training set (60%), a validation set (15%), and a testing set (25%) in each fold.

The multi-omics data usually suffer from the “curse of dimensionality,” where the number of features is significantly larger than the number of samples. To mitigate this challenge, we apply feature selection or dimension reduction techniques to get rid of the unrelated or redundant features, which are essential for the success of downstream analysis such as classification or survival analysis. For classification, supervised univariate feature selection methods such as minimum Redundancy Maximum Relevance (mRMR) [21] and mutual information can be used. For survival analysis, various unsupervised or knowledge-guided feature selection can be applied. For example, Huang et al. have applied gene co-expression analysis as the dimension reduction approach [19]. In this study, with the focus on deep-learning based feature-level integration, we use both principal component analysis (PCA) and unsupervised variance-based feature selection. In PCA-based dimension reduction, we apply PCA to the training dataset and use the first 100 principal components (PCs) of training, validation, and testing datasets for survival analysis. In unsupervised variance-based feature selection, we select the top 1000 features with the highest variances from the training dataset, and then use them for survival analysis in training, validation, and testing datasets.

### Single-modality network

**Fig. 3:**
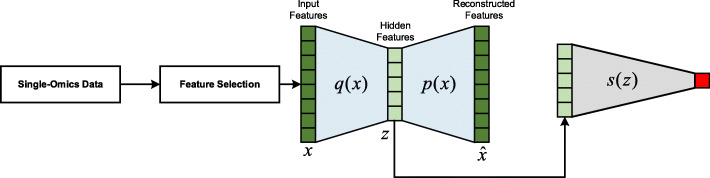
Single-omics data survival analysis network. The input data *x* is represented with an encoder *q*(*x*) into hidden feature *z* and then constructed with a decoder *p*(*x*). We then feed the hidden feature *z* into a task-specific network such as multi-class classification or survival analysis

For single-modality data, we use an autoencoder and a task-specific network for single-modality classification or survival analysis (Fig. 3). For the input data *x* after feature selection, we first apply an encoder *q*(*x*) to transform the input data to a hidden feature *z*, and then reconstruct the input data $\hat{x}$ from the hidden feature with a decoder *p*(*z*). We then feed the hidden feature *z* into a task-specific network for classification or survival analysis.

#### Endpoint 1: multi-class classification

For the classification network *c*(*z*), we use a fully connected network with the output dimension size the same as the number of classes. Thus, the whole network is trained with the reconstruction loss *L*_*recon*_ and the classification loss *L*_*cls*_. In this study, we use the mean-square error for the reconstruction loss:

$$ {L}_{recon}=\frac{1}{N}\sum \limits_1^N{\left({x}_n-{\hat{x}}_n\right)}^2 $$

where *N* is the batch size. We use the cross-entropy loss for the classification loss:

$$ {L}_{clf}=-\log \left(\frac{\exp \left(x\left[ class\right]\right)}{\sum_{j=1}^C\left(x\left[j\right]\right)}\right)=-x\left[ class\right]+\log \left(\sum \limits_{j=1}^C\left(x\left[j\right]\right)\right) $$

where *C* is the number of classes and *j* ∈ {1, …, *C*}. For each epoch, we first train the encoder-decoder with the reconstruction loss *L*_*recon*_ and then train the encoder and classification network with the cross-entropy loss *L*_*clf*_.

The multi-class classification performance is evaluated by accuracy, weighted precision, and weighted recall. These metrics are in the range of [0, 1], and the higher the better. We do not include AUC as a metric because we perform 10-class classification with the simulated MNIST dataset instead of binary classification.

#### Endpoint 2: survival analysis

For the survival analysis, we use a fully connected neural network *s*(*z*), to replace the Cox proportional hazards model. The output of the survival network *s*(*z*) is the hazard *h* of the patient. Based on the Cox proportional hazards model, the survival network is trained with the negative log partial likelihood loss *L*_*sur*_:

$$ {L}_{sur}=-\frac{1}{N_{ob}}\sum \limits_{i:{C}_i=1}\left({h}_i-\log \sum \limits_{j:{T}_j\ge {T}_i}\exp \left({h}_j\right)\right) $$

Where *C*_*i*_ = 1 indicates the occurrence of the event for patient *i*, *N*_*ob*_ is the total number of events in the batch, and *T*_*i*_ and *T*_*j*_ are the survival time for patient *i* and patient *j*, respectively.

To evaluate the risk scores predicted by survival models, various metrics have been developed to measure the concordance between the predicted risk scores and the actual survival time. Following the previous studies in deep-learning-based survival analysis [19], we evaluate the overall survival analysis performance with the concordance index (C-index) [22]. C-index evaluates how well the survival risk we computed aligns with the actual survival time given any two comparable pairs:

$$ \mathrm{C}-\mathrm{index}=\Pr \left\{{h}_i>{h}_j|{T}_i<{T}_j,{C}_i=1\right\} $$

### Novel multi-modality integration network

We develop novel multi-omics integration networks based on two principles in multi-view machine learning: 1) the complementary principle assumes that each view contains information other views do not have, and we should extract the difference from each view while preserving the common information; and 2) the consensus principle assumes that the disagreements between views upper bound the classification errors; thus, we should aim to maximize the agreement between views. Based on these principles, we have used this novel strategy to learn meaningful representations by integrating data from multiple modalities.

#### Integrating the complementary information: concatenation autoencoder (ConcatAE)

**Fig. 4:**
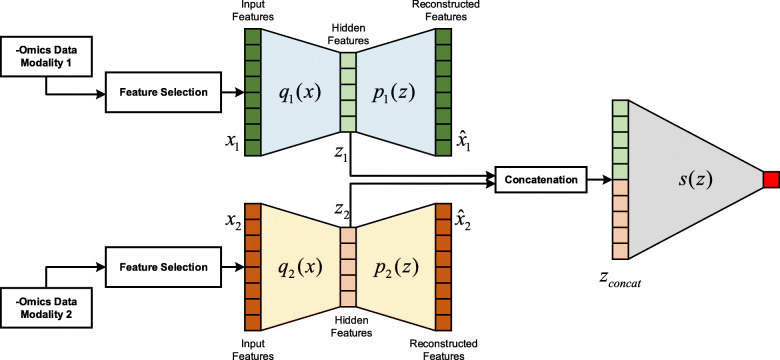
Multi-omics data integration with concatenation autoencoder (ConcatAE). The hidden features of each data modality are concatenated before feeding into the task-specific network

We use the concatenation autoencoder (ConcatAE) to integrate the complementary information from each data modality (Fig. 4). For each modality, we train an independent autoencoder and transform the input features into a hidden space. We then concatenate the hidden features from each modality and feed the concatenated hidden feature into the task-specific network. Compared to the single-modality network, we have a separate reconstruction loss for each data modality. Thus, the reconstruction loss is the summation of these separate reconstruction losses. For example, when integrating two modalities, the new reconstruction loss would be:

$$ {L}_{recon}^{\prime }=\frac{1}{N}\sum \limits_1^N\left({\left({x}_{1,n}-{\hat{x}}_{1,n}\right)}^2+{\left({x}_{2,n}-{\hat{x}}_{2,n}\right)}^2\right) $$

The task-specific network training procedure remains the same, with the input becoming the concatenation of hidden features represented from each modality.

#### Integrating the consensus information: cross-modality autoencoder (CrossAE)

**Fig. 5:**
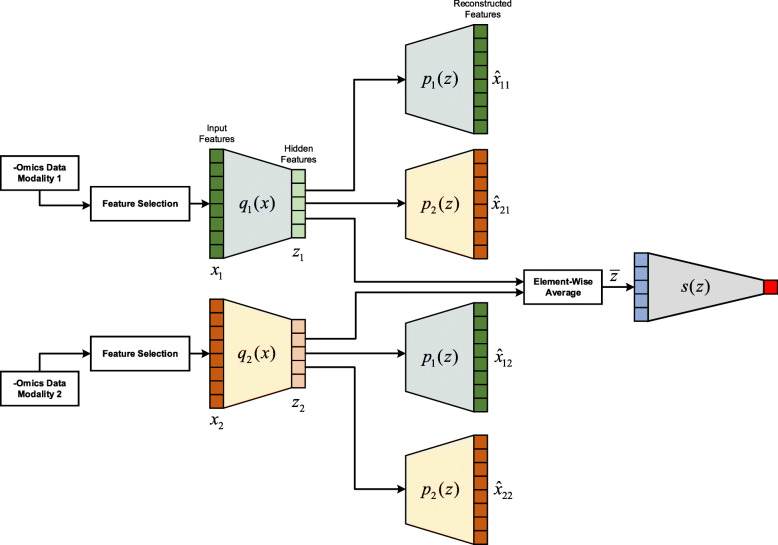
Multi-omics data integration with cross-modality autoencoder (CrossAE). For hidden features of each data modality, they are used to reconstruct input features of both the original modality and other modalities. The hidden features of various modalities are element-wise averaged before feeding into the task-specific network

We use the cross-modality autoencoder (CrossAE) to integrate the consensus information from each data modality (Fig. 5) through cross-modality translation. To enable consensus representation among modalities, it uses the hidden features extracted from one modality to reconstruct the input features from other modalities.

We train the framework with three steps. In the first step, we train an autoencoder for each modality independently, as we have done in the ConcatAE model with ${L}_{recon}^{\prime }$. In the second step, we train these encoders and decoders again with cross-modality reconstruction. For example, the modality 1 encoder *q*_1_(*x*) is used to transform input data *x*_1_ to hidden feature *z*_1_ = *q*_1_(*x*_1_). We then use the modality 2 decoder *p*_2_(*z*) to reconstruct the modality 2 input data *x*_2_ from *z*_1_, which is denoted as ${\hat{x}}_{21}={p}_2\left({z}_1\right)$. We can perform similar cross-modality reconstruction from modality 2 hidden features *z*_2_ to modality 1 input data *x*_1_. Thus, the cross-modality reconstruction loss *L*_*cross* _ *recon*_ for step 2 with two modalities is

$$ {L}_{cross\_ recon}=\frac{1}{N}\sum \limits_1^N\left({\left({x}_{1,n}-{\hat{x}}_{12,n}\right)}^2+{\left({x}_{2,n}-{\hat{x}}_{21,n}\right)}^2\right) $$

In the third step, we combine the hidden features from each modality with the element-wise average and then train the encoders and task-specific network with task-specific loss (e.g., the cross-entropy loss for classification or the negative partial log-likelihood loss for survival regression). We implemented and tested the proposed integration models on two data modalities. These frameworks can be naturally extended to the integration of more than two data modalities.

### Implementation and experiments

The train-test split for cross-validation and the classification metrics are implemented with [23]. The neural networks are designed and implemented with PyTorch 1.1.0. For cancer type classification, we use a batch size of 32, and Adam optimizer with a learning rate of 0.001, and training epochs of 200. For survival analysis, we use a batch size of 128, and Adam optimizer with a learning rate of 0.001, and training epochs of 200. More details of the model implementation and training details can be found at Github repo (https://github.com/tongli1210/BreastCancerSurvivalIntegration).

## Results

### Multi-modality integration simulation

We first test the proposed single and multi-modal integration networks on the simulated MNIST datasets (*S*_1_ and *S*_2_). The results are presented in Table 2. From the results, we observe significant classification performance improvements after multi-modality data integration for both random erasing dataset *S*_1_ and the Gaussian noise erasing dataset *S*_2_. For dataset *S*_1_, we assume the model should take the complementary information from *X*_1_ and *X*_2_ to get better performance. From the experiment results, the integration model ConcatAE does perform slightly better compared to the integration model CrossAE. For dataset *S*_2_, because of the global noises for both views, we assume the model should take the consensus information from *S*_1_ and *S*_2_ to get better performance. From the experiment results, we observe CrossAE achieves better performance compared to ConcatAE, which is as expected.

**Table 2:** Multi-modality integration simulation with MNIST dataset

**Modalities**	**Random Erasing (*S***_**1**_**)**			**Gaussian Noise (*S***_**2**_**)**		
	**ACC**	**Precision**	**Recall**	**ACC**	**Precision**	**Recall**
**X1**	0.942 ± 0.004	0.942 ± 0.004	0.942 ± 0.004	0.884 ± 0.003	0.886 ± 0.003	0.884 ± 0.003
**X2**	0.942 ± 0.003	0.943 ± 0.003	0.942 ± 0.003	0.879 ± 0.005	0.881 ± 0.005	0.879 ± 0.005
**ConcatAE(X1 + X2)**	**0.962 ± 0.001**	**0.963 ± 0.001**	**0.962 ± 0.001**	0.924 ± 0.001	0.925 ± 0.002	0.924 ± 0.001
**CrossAE(X1 + X2)**	0.962 ± 0.002	0.962 ± 0.002	0.962 ± 0.002	**0.933 ± 0.002**	**0.933 ± 0.002**	**0.933 ± 0.002**

### Multi-modality integration for breast cancer survival analysis

The performance of the single-omics survival analysis model is presented in Table 3. We observe that the model achieves better performance when using PCA features compared with that using the high variance features for all modalities except for CNVs. Among the four -omics data, miRNA expression is the most predictive for overall survival, followed by DNA methylation and gene expression. Moreover, CNVs are the least predictive for breast cancer overall survival, which is consistent with our previous findings [20]. The best single-omics survival analysis performance is a C-index of 0.616 ± 0.057, achieved by miRNA data with PCA features.

**Table 3:** Performance of single-omics survival analysis model

**Data Modality**	**Gene Expression**	**DNA Methylation**	**miRNA Expression**	**Copy Number Variation**
**PCA**	0.589 ± 0.084	0.583 ± 0.058	**0.616 ± 0.057**	0.476 ± 0.051
**Variance**	0.529 ± 0.033	0.581 ± 0.066	0.614 ± 0.041	0.503 ± 0.071

The performance of the novel multi-omics integration survival analysis model is presented in Table 4. Based on the results, we observe that integration is not always beneficial for performance. For example, the integration of gene expression and DNA methylation high variance features can lead to lower C-index (0.507 ± 0.036) than either gene expression (0.529 ± 0.033) or DNA methylation (0.581 ± 0.066) alone. Among the six combinations of two-omics data integration, we found the integration of DNA methylation and miRNA expression consistently achieves a good performance. Comparing the two integration strategies, we found that the ConcatAE outperforms the CrossAE in most experiments. Comparing the two feature selection strategies, we observed that the PCA features outperform high variance features in most experiments except for those involves CNV data. We believe the PCA dimension reduction approach may not be suitable for the discrete CNV data. Among all multi-omics integration models, the best performance (0.641 ± 0.031) is achieved by integrating DNA methylation and miRNA expression using PCA features and the ConcatAE model.

**Table 4:** Performance of multi-omics survival analysis model

**Integration**	**Data Modality**	**GeneExp** **+** **DnaMeth**	**GeneExp** **+** **miRNA**	**GeneExp** **+** **CNVs**	**DnaMeth** **+** **miRNA**	**DnaMeth** **+** **CNVs**	**miRNA** **+** **CNVs**
**ConcatAE**	**PCA**	0.585 ± 0.107	0.59 ± 0.093	0.576 ± 0.047	**0.641 ± 0.031**	0.583 ± 0.09	0.588 ± 0.057
	**Variance**	0.507 ± 0.036	0.53 ± 0.052	0.524 ± 0.038	0.625 ± 0.023	0.586 ± 0.068	0.603 ± 0.04
**CrossAE**	**PCA**	0.583 ± 0.07	0.595 ± 0.062	0.553 ± 0.045	0.63 ± 0.081	0.579 ± 0.065	0.578 ± 0.028
	**Variance**	0.511 ± 0.027	0.558 ± 0.054	0.53 ± 0.033	0.605 ± 0.059	0.576 ± 0.026	0.613 ± 0.066

**Fig. 6:**
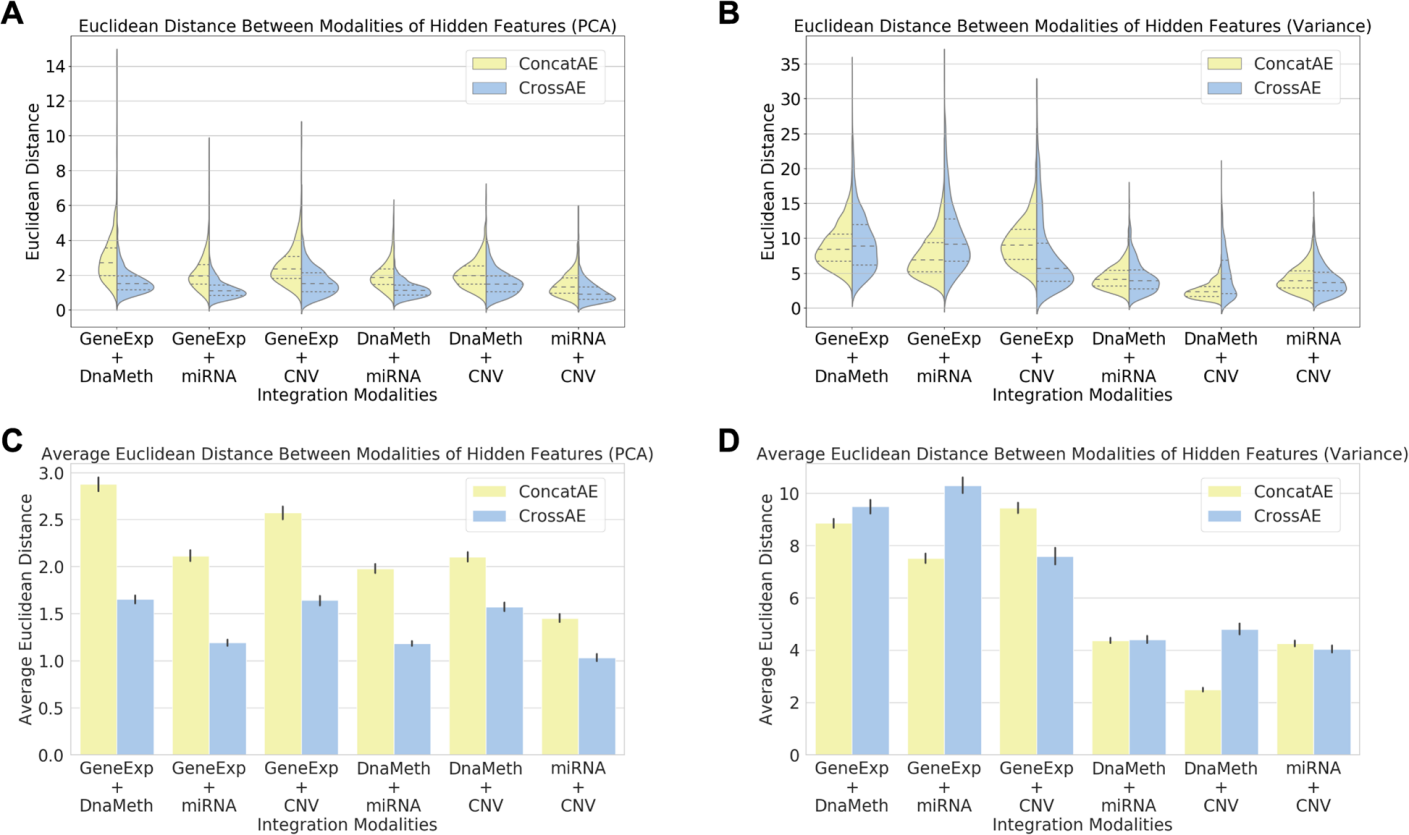
Similarity measure with Euclidean distance of the paired hidden features. We measure the similarity of paired hidden features with the Euclidean distance. **a** Grouped violin plots of the Euclidean distances for hidden features represented from PCA features. **b** Grouped violin plots of the Euclidean distances for hidden features represented from high variance features. **c** Grouped bar plots of the average Euclidean distances for hidden features represented form PCA features. **d** Grouped bar plots of the average Euclidean distances for hidden features represented from high variance features. Yellow: ConcatAE. Blue: CrossAE

To evaluate the consensus among hidden features, we measure the similarity of paired hidden features with the Euclidean distance, and visualize their distributions with grouped violin plots in Fig. 6. The violin plots are grouped by multi-omics modalities under integration (e.g., GeneExp+miRNA) and compared for the two integration methods ConcatAE and CrossAE. For the hidden features (dimension of 10) represented from PCA features, we can observe higher similarities (or lower Euclidean distances) for integration using CrossAE compared to those using ConcatAE (Fig. 6a). However, for the hidden features (dimension of 100) represented from high variance features, the CrossAE method will not necessarily lead to higher similarities (Fig. 6b). The observation is further confirmed with grouped bar plots of the average Euclidean distances in Fig. 6c and d. The results indicate that the consensus constraints imposed by CrossAE work well for PCA features but suffer for the high variance features, which has a much higher dimension.

To further understand the similarity between paired hidden features, we tried to use the t-Distributed Stochastic Neighbor Embedding (t-SNE) to visualize the hidden features from the first fold of our four-fold cross-validation in the Supplementary Material Section S3. If using 100 PCA features as the input data, we observe more overlap among the CrossAE hidden features (Green and Yellow) than the ConcatAE hidden features (Red and Blue) (See Fig. S2). This indicates that the multi-omics data representation by CrossAE is more complied with consensus constraints. However, if using 1000 high variance features as the input data, we observe that the distribution patterns of the ConcatAE hidden features (Red and Blue) are similar to those of the CrossAE hidden features (Green and Yellow) (See Fig. S3). This implies that the effect of consensus constraints by CrossAE is not as significant.

## Discussions

In this study, we have developed two novel multi-modal data integration strategies: to integrate the complementary information among modalities with ConcatAE; and to integrate the consensus information using CrossAE. We have tested the two new models on the simulated MNIST data and validated their effectiveness. We then apply the two new models to the multi-omics breast cancer survival data. ConcatAE model integrating DNA methylation and miRNA expression PCA features achieves the best performance with a C-index of 0.641 ± 0.031 and outperforms that of the CrossAE model (0.63 ± 0.081). Both integration approaches outperform the corresponding single-modality model, which uses DNA methylation or miRNA expression alone. The results indicate that these two modalities should have both complementary and consensus information for survival prediction.

Although the ConcatAE outperforms CrossAE, we believe this does not necessarily indicate that the complementary information is more important than the consensus information. As we have seen in the MNIST simulated data with Gaussian noise, if the multi-modality data are noisy and equally predictive, consensus learning can achieve higher prediction performance compared to that of complementary learning. Moreover, the ConcatAE model should include both the modality-invariant and modality-unique information, although neither has been specifically maximized.

The best survival prediction performance is achieved by integrating DNA methylation and miRNA expression PCA features. However, the results are insufficient to conclude that DNA methylation or miRNA expression is more informative than the other modalities. Due to the lack of biological ground-truth, the model interpretation and wet-lab validation are needed to understand the model. As a black-box model, we cannot currently locate which biomarkers (e.g., specific genes or methylation sites) are picked by the integration network and contribute more to the final survival prediction. Thus, as a future direction, we propose to apply model interpretation methods to the deep network and to validate the biomarkers by literature or by wet-lab experiments. Such validation can provide insight into why some integration models outperform the others and is critical for translation to clinical practice.

Although we have demonstrated the effectiveness of ConcatAE and CrossAE for multi-omics data integration in this study, future improvements can be made in the following three areas: 1) training data, 2) model validation, and 3) model improvements.

The first improvement is on the training data, which dictates the survival prediction performance. For example, in the TCGA-BRCA dataset, the CNV features are the least predictive for breast cancer survival. One potential cause is that the CNV features from the TCGA database are categorical (i.e., “gain”, “loss”, or “normal”) and might constrain the predictive capability of this modality. In addition, the gene expression data are normalized with FPKM and the miRNA expression data are normalized with RPM. FPKM and RPM normalization are potentially biased when comparing between samples. The survival prediction performance can be further improved for gene expression and miRNA expression if replacing the normalization method with more sophisticated bioinformatics techniques such as transcripts per million (TPM). Another essential limitation of the current training dataset is the relatively small sample size of the TCGA-BRCA dataset with around 1000 patients. For a data-driven approach, the performance of deep learning is significantly influenced by the amount of training data. One future direction is to improve our model by using a larger breast cancer survival dataset or by combining multi-source breast cancer survival datasets. Another future direction is to make the most of the TCGA database by multi-task learning, such as applying the integration methods to cancer staging, subtyping, and grading in addition to survival analysis.

The second limitation is model validation. In this study, we validate the effectiveness of ConcatAE and CrossAE networks with the simulated two-view imaging data from the MNIST database, in which we have controlled and visualized the consensus and complementary information. Ideally, a cancer genomics dataset with ground truth would be preferred to validate the proposed integration networks. However, to the best of our knowledge, there is no such golden standard multi-omics dataset developed yet because many complex interactions among multi-omics data remain unknown. If the ground-truth of multi-omic interactions were known, it would be straightforward to validate the consensus and complementary principles for multi-omics data integration methods. Before it happens, a more realistic approach is to collect data for the known cross-modality pathways (e.g., DNA methylation and gene expression pathways) to validate the consensus principle. Another way is to use the multi-omics data simulation with ground truth to validate the proposed models. Although some multi-omics data simulation works have been recently developed [24, 25], they are not specifically designed to validate the interactions across modalities with 1) consensus information (e.g., co-regulation pathways), 2) complementary information (e.g., modality-specific pathways/biomarkers), and 3) endpoint irrelevant information. Thus, one promising future step is to simulate multi-omics data to validate the integration principles and methods in the follow-up studies.

The third limitation lies in the multi-modality integration network. First, we have shown that the feature-selection or dimension-reduction steps impact multi-modality integration performance. Our current feature selection step contains unsupervised feature selection by variance ranking and unsupervised dimension reduction by PCA. One immediate future work is to utilize more sophisticated knowledge-guided feature selection. Another future work is to integrate feature selection with multi-omics feature representation into the multi-modality deep network to improve model performance. Second, combining consensus learning and complementary learning may further improve multi-omics integration. We propose to extend the current ConcatAE framework by using two encoders or an encoder with branches to represent both the modality-unique hidden feature and the modality-consensus feature. The modality-unique hidden features can be learned by maximizing the divergence among modalities, while the modality-consensus hidden features can be learned by minimizing the divergence among modalities. Instead of cross-modality reconstruction in CrossAE, the consensus constraints and the complementary constraints are both realized by divergence optimization for better performance. Third, another future direction is to improve the survival model. In this study, we have implemented a simple deep learning-based survival network using the negative partial log-likelihood loss. One future work is to improve the survival network with regularization, such as *L*_1_ loss on the network weights. A robust survival network will further improve the multi-omics integrated survival network.

## Conclusions

In this study, we have investigated two novel multi-modal data integration strategies: ConcatAE and CrossAE. We first tested the proposed models on the simulated MNIST data and validated the effectiveness of ConcatAE in integrating complementary information and CrossAE in integrating consensus information among multi-modality data. We then apply the proposed models to the multi-omics breast cancer survival data obtained from the TCGA-BRCA dataset. For the single-omics model, the miRNA expression is the most predictive for breast cancer survival analysis (0.616 ± 0.057), followed by DNA methylation and gene expression. CNV data is the least predictive for breast cancer overall survival analysis. For the multi-omics model, the ConcatAE model integrating DNA methylation and miRNA expression PCA features achieves the best performance with a C-index of 0.641 ± 0.031. The CrossAE model integrating DNA methylation and miRNA expression PCA features achieves a C-index of 0.63 ± 0.081, which also outperforms either DNA methylation or miRNA expression alone. We conclude that the DNA methylation data and miRNA expression data contain both complementary and consensus information, and using such information can improve survival analysis performance. As a future direction, we can develop a sophisticated learning framework utilizing both consensus and complementary information simultaneously to further improve survival prediction for personalized breast cancer diagnosis and treatment.

## Supplementary information

**Additional file 1.** Supplementary file and supplementary figures.

## Data Availability

The MNIST database can be accessed at http://yann.lecun.com/exdb/mnist/ The TCGA-BRCA breast cancer multi-omics data can be downloaded from https://portal.gdc.cancer.gov/

## Abbreviations

AUC: Area under the curve
BRCA: BReast CAncer
C-index: Concordance index
CNVs: Copy number variations
ConcatAE: Concatenation autoencoder
CrossAE: Cross-modality autoencoder
FPKM: Fragments per kilobase of transcript per million mapped reads
MNIST: Modified national institute of standards and technology
mRMR: minimum Redundancy Maximum Relevance
PCA: Principal component analysis
PCs: Principal components
RPM: Reads per million mapped reads
TCGA: The cancer genome atlas
TPM: Transcripts per million
