# Supplementary material for: Deep learning based feature-level integration of multi-omics data for breast cancer patients survival analysis
Source: BMC Med Inform Decis Mak. 2020 Sep 15;20:225. doi: 10.1186/s12911-020-01225-8 (PMC7493161; doi:10.1186/s12911-020-01225-8)
Supplement: Supplementary file 1 — Additional file 1. Supplementary file and supplementary figures. [file 12911_2020_1225_MOESM1_ESM.docx]

# S1. TCGA Multi-Omics Data

The breast cancer multi-omics data in this study are obtained from The Cancer Genome Atlas (TCGA) [1]. For each -omics modality (i.e., gene expression, DNA methylation, miRNA expression, and copy number variation (CNV)), the bioinformatics pipelines for feature extraction are performed by TCGA. We obtained the preprocessed multi-omics tabular data from the TCGA data portal (<https://portal.gdc.cancer.gov/>).

Here are the TCGA documentation of the four multi-omics data, respectively:

1) Gene Expression (mRNA Expression):

<https://docs.gdc.cancer.gov/Data/Bioinformatics_Pipelines/Expression_mRNA_Pipeline/>

The gene expression table we used is the FPKM-UQ (upper quartile normalization) normalized gene expression. Based on our investigation, the gene expression table has 60,483 features, which are different isoforms for each gene and some non-coding RNA transcripts.

2) DNA methylation:

<https://docs.gdc.cancer.gov/Data/Bioinformatics_Pipelines/Methylation_LO_Pipeline/>

The DNA methylation data we used is the methylation array, which is a table of beta values. The data are processed from Illumina human methylation 450 or Illumina human methylation 27 assays.

3) miRNA expression

<https://docs.gdc.cancer.gov/Data/Bioinformatics_Pipelines/miRNA_Pipeline/>

The miRNA data we used is the miRNA expression quantification table. The expression table is generated by TCGA with a modified profiling pipeline developed by the British Columbia Genome Sciences Centre (BCGSC).

4) Copy number variation (CNV)

<https://docs.gdc.cancer.gov/Data/Bioinformatics_Pipelines/CNV_Pipeline/>

The copy number variation we used is the “Gene Level Copy Number Scores” table. The table contains CNV scores represented with gains/losses on a gene level for all samples in the TCGA-BRCA project.

# S2. Subject Intersection

After preprocessing, the TCGA-BRCA multi-omics dataset contains gene expression data for 1,092 subjects, DNA methylation data for 1,095 subjects, miRNA data for 1,079 subjects, CNV samples for 1,089 subjects, and survival data for 1095 subjects. Some of the subjects have more than one -omics samples; we have randomly selected one sample for these patients.

As we aim for multi-omics integration, we remove the samples with missing modalities and keep only the samples with all four -omics data (i.e., mRNA, miRNA, DNA methylation, and CNVs) for survival analysis. After performing this step, we have 1,060 breast cancer subjects (patients). All these 1,060 patients are breast cancer patients with survival information (survival time and event). The intersections of these subjects are visualized with the Venn diagram below in Figure S1.


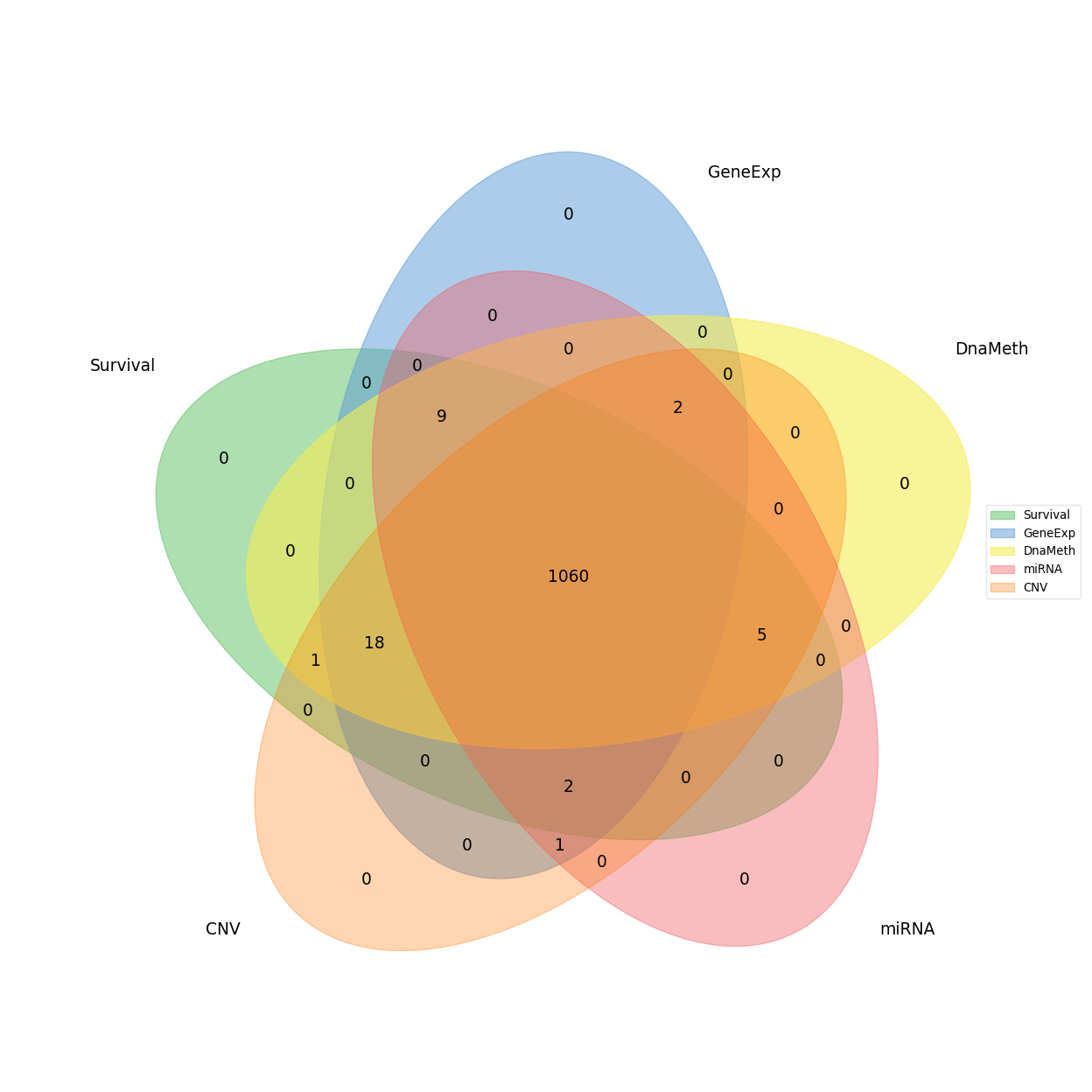


Figure S1. Venn Diagram of the subject intersections in the TCGA-BRCA multi-omics dataset.

# S3. t-SNE Visualization of Hidden Features

We use the t-Distributed Stochastic Neighbor Embedding (t-SNE) to visualize the hidden features represented from each -omics modality. The t-SNE is a dimension reduction technique usually applied for the visualization of high-dimensional data [2]. We have applied the t-SNE to hidden features of the first fold of our four-fold cross-validation and to PCA features/high variance features, respectively.

**Figure S2** is the t-SNE visualization of the hidden features represented from PCA features using either ConcatAE or CrossAE. The dimension of these hidden features is 10 before applying t-SNE. Based on the t-SNE visualization, we can observe better overlaps of the CrossAE features (Green and Yellow) compared to those of the ConcatAE features (Red and Blue), which indicate the effect of consensus constraints on multi-omics data representation.


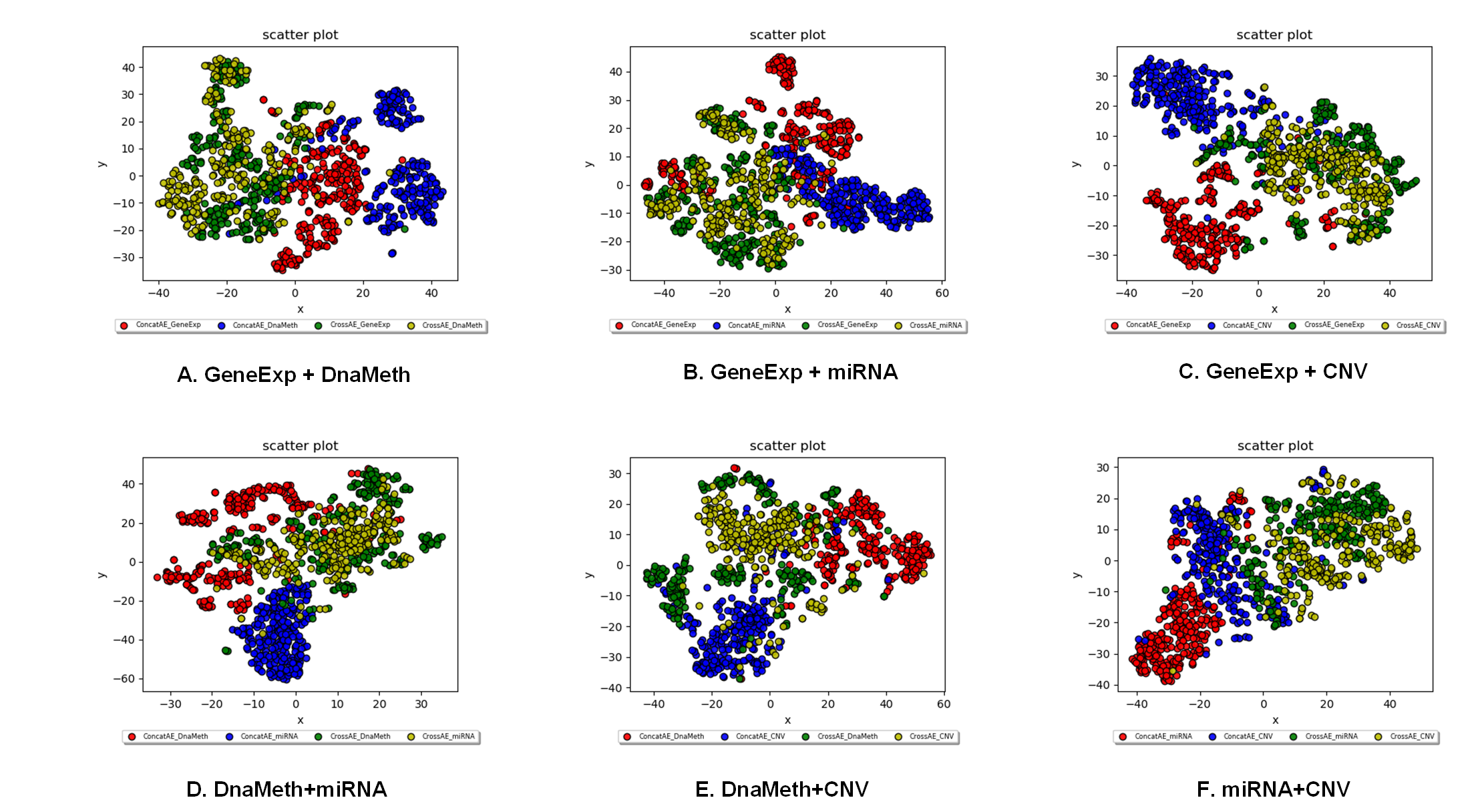


Figure S2. t-SNE visualization of the hidden features, represented from the PCA features.

# S3. t-SNE Visualization of Hidden Features (Cont’d)

**Figure S3** is the t-SNE visualization of the hidden features represented from high variance features using either ConcatAE or CrossAE. The dimension of these hidden features is 100 before applying t-SNE. Based on the t-SNE visualization, we observe similar patterns for the ConcatAE features (Red and Blue) and the CrossAE features (Green and Yellow). For the high variance features, the effect of consensus constraints by CrossAE is not significant. For example, we can observe a closer geographical relationship for the CrossAE features compared to that of ConcatAE features only in **Figure S3D**.


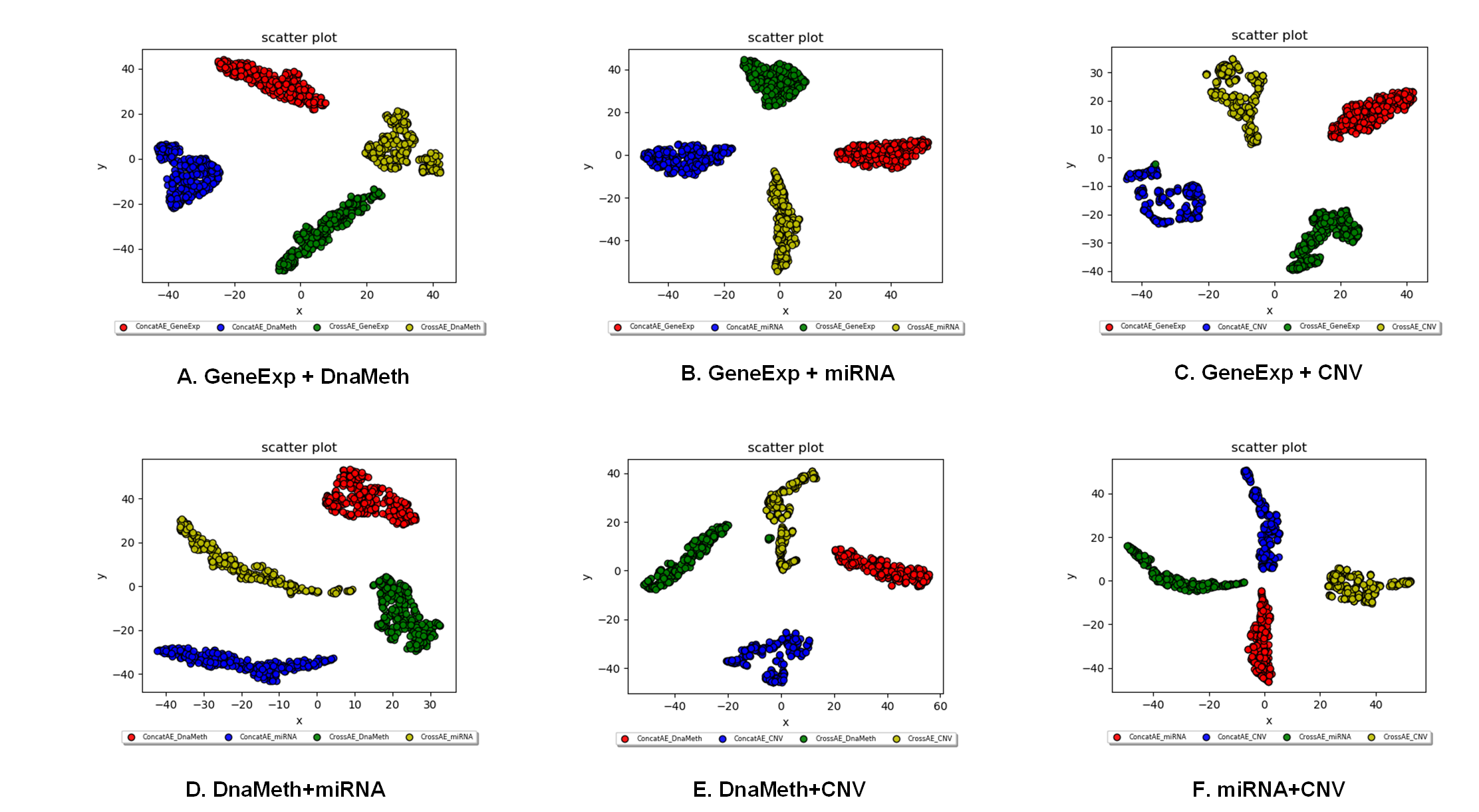


Figure S3. t-SNE visualization of the hidden features, represented from the high variance features.

# References

1. Grossman RL, Heath AP, Ferretti V, Varmus HE, Lowy DR, Kibbe WA, Staudt LM: **Toward a shared vision for cancer genomic data**. *New England Journal of Medicine* 2016, **375**(12):1109-1112 %@ 0028-4793.

2. Maaten Lvd, Hinton G: **Visualizing data using t-SNE**. *Journal of machine learning research* 2008, **9**(Nov):2579-2605.
